# Ablation of PGC1 beta prevents mTOR dependent endoplasmic reticulum stress response

**DOI:** 10.1016/j.expneurol.2012.06.031

**Published:** 2012-10

**Authors:** Alberto Camacho, Sergio Rodriguez-Cuenca, Margaret Blount, Xavier Prieur, Nuria Barbarroja, Maria Fuller, Giles E. Hardingham, Antonio Vidal-Puig

**Affiliations:** a University of Cambridge Metabolic Research Laboratories, NIHR Cambridge Biomedical Research Centre Institute of Metabolic Science, Addenbrooke's Hospital, Cambridge, UK; b Hospital Virgen de la Victoria, Málaga, CIBER Fisiopatología de la Obesidad y Nutrición, Instituto de Salud Carlos III, Spain; c University of Edinburgh, Centre for Integrative Physiology, Edinburgh, UK; d Lysosomal Diseases Research Unit, SA Pathology at Women's and Children's Hospital, North Adelaide, SA 5006, Australia

**Keywords:** PGC 1 beta, Endoplasmic reticulum stress, Mitochondria, mTOR, Amino acids, Brain

## Abstract

Mitochondria dysfunction contributes to the pathophysiology of obesity, diabetes, neurodegeneration and ageing. The peroxisome proliferator-activated receptor-gamma coactivator-1β (PGC-1β) coordinates mitochondrial biogenesis and function as well as fatty acid metabolism. It has been suggested that endoplasmic reticulum (ER) stress may be one of the mechanisms linking mitochondrial dysfunction and these pathologies. Here we investigate whether PGC-1β ablation affects the ER stress response induced by specific nutritional and pharmacological challenges in the CNS. By using flow cytometry, western blot, real time PCR and several pharmacological and nutritional interventions in PGC-1β knock out and WT mice, we confirmed that PGC-1β coordinates mitochondria function in brain and reported for the first time that a) ablation of PGC-1β is associated with constitutive activation of mTORC1 pathway associated with increased basal GRP78 protein levels in hypothalamus and cortex of animals fed chow diet; and b) in animals fed chronically with high fat diet (HFD) or high protein diet (HPD), we observed a failure to appropriately induce ER stress response in the absence of PGC-1β, associated with an increase in mTOR pathway phosphorylation. This contrasted with the appropriate upregulation of ER stress response observed in wild type littermates. Additionally, inefficient *in vitro* induction of ER stress by thapsigargin seems result in apoptotic neuronal cell death in PGC-1β KO. Our data indicate that PGC-1β is required for a neuronal ER response to nutritional stress imposed by HFD and HPD diets and that genetic ablation of PGC-1β might increase the susceptibility to neuronal damage and cell death.

## Highlights

► The PGC-1β coordinates mitochondrial function and fatty acid metabolism. ► Ablation of PGC-1β associates with mTORC1 activation and basal increase of GRP78. ► Metabolic stress results in inefficient GRP78 increase in PGC-1β KO mice.

## Introduction

Maintenance of energy homeostasis is coordinated by the crosstalk between peripheral organs and the central nervous system and more specifically the hypothalamus (Stefater and Seeley, 2010). Glucose, lipids and amino acids are metabolized in the mitochondria and defects in mitochondrial function may contribute to nutrient overload causing cellular damage as typically associated with obesity, insulin resistance, cardiovascular disease, neurodegeneration or accelerated ageing (Mattson et al., 2008, Patti and Corvera, 2010).

Peroxisome-proliferator-activated-receptor γ coactivator 1 beta (PGC-1β) regulates mitochondrial biogenesis and function through its coactivating effects on specific nuclear receptors (Tseng, et al., 2010). PGC-1β is highly expressed in pro-oxidative tissues such as heart, skeletal muscle, and brown fat but also in the brain (Lin et al., 2002, Meirhaeghe et al., 2003). There is evidence that PGC-1β genetic ablation results in defective mitochondrial activity leading to defects in thermogenesis, hepatic function, and cardiac performance (Lelliott et al., 2006, Lelliott et al., 2007, Sonoda et al., 2007). Of note, the PGC-1β KO mice are viable and apparently metabolically healthy when fed a chow diet, however, when challenged with an acute load of high fat diet (HFD), they develop liver steatosis (Lelliott et al., 2006, Sonoda et al., 2007), suggesting that PGC-1β is required for the appropriate regulation of lipid metabolism, particularly under stressful metabolic conditions (Lelliott et al., 2006, Sonoda et al., 2007).

Mammalian target of rapamycin (mTOR) core is a serine/threonine kinase comprised of two distinct protein complexes referred to as TORC1 and TORC2 (Howell and Manning). TORC1 complex is composed of the mTOR, mLST8, raptor and deptor, and its main function is to sense changes in amino acids, glucose and oxygen availability while promoting adaptive cell growth responses mediated by phosphorylation of 4E-BP1 and S6K1/2 proteins (Zoncu, et al.). It has recently been demonstrated that nutrients such as glucose and amino acids exert a direct role promoting mTORC1 activation (Inoki et al., 2003, Kim et al., 2002). Interestingly, mTORC1 activates basal mitochondrial function and biogenesis (Cunningham et al., 2007, Schieke et al., 2006), whereas its disruption may result in cell damage in many disorders (Bentzinger et al., 2008, Risson et al., 2009). However, chronic mTORC1 activation may lead to metabolic derangements and pathologic states (Weichhart, 2012). Of note, mTORC1 is physically associated with the mitochondria through the FKBP12-rapamycin associated protein (FRAP) (Desai, et al., 2002); however, there is little knowledge about the molecular mechanism involved in such interaction and its cellular effects during chronic mTORC1 stimulation. Of interest, mTOR may also activate endoplasmic reticulum (ER) stress response (Di Nardo et al., 2009, Ozcan et al., 2008) and indeed its activation has been demonstrated in several disorders such as diabetes, cardiovascular disease, cancer, neurodegeneration and aging (Tsang et al., 2007, Zoncu et al., 2011). This suggests that mTOR may be a functional node linking mitochondrial dysfunction and ER stress susceptibility and thus influencing the development of metabolic disturbances. However, it is unclear whether this may be the cause or consequence of mitochondrial dysfunction.

Here we investigate the link between defects in mitochondria function induced by PGC-1β ablation, the activation of mTORC1 and the induction of an ER stress response. Moreover, we investigated whether under specific nutritional stressors such as HFD and/or high protein diet (HPD), the absence of PGC-1β results in increased vulnerability to neuronal death.

## Materials and methods

### Animal models

All the experiments were performed using wild-type and PGC-1β KO on a C57BL/6 background generated as previously reported (Lelliott, et al., 2006). Animal procedures were performed in accordance with the UK Home Office regulations and the UK Animal Scientific Procedures Act [A(sp)A 1986. Animals were housed in a temperature-controlled room with a 12-h light/dark cycle. Food and water were available *ad libitum*.

### Immunoblotting

Brain regions (including hypothalamus and cortex) were dissected and homogenized in lysis buffer as described (Camacho, et al., 2007). Immunoblotting was performed following the protocol previously published with minor modifications (Camacho, et al., 2007). The following antibodies were used (Suppl. Fig. 1).

### Mice, long-term feeding and treatments

Mice were placed on three different diets: 1) at weaning (4-week-old) on normal chow diet (4.8% fat, 74.3% carbohydrates, and 16.8% protein with a total energy content of 4.07 kcal/g) (R3 diet, Lactamin AB, Stockholm, Sweden); 2) at weaning (4-week-old) on High Fat Diet (HFD, 45% fat, 35% carbohydrates and 20% protein, total energy 4.73 kcal/g) (D12451, Research diets, New Jersey, USA) during 24 weeks; and 3) at 6 months on High Protein Diet (HPD) (4.3% fat, 48.1% carbohydrates, and 38.5% protein with a total energy content of 3.85 kcal/g) (D03091001, Research diets, New Jersey, USA) during 14 weeks.

### *In vivo* ER stress induction

Wild-type and PGC-1β KO were given a single intraperitoneal injection of 1 μg/g body weight of tunicamycin as previously reported (Zhang and Kaufman, 2008). 24 h after the injection, the mice were sacrificed and the brain regions were processed for western blot or real time PCR analysis as described below.

### RNA extraction and real-time PCR

Brain tissues used for RNA extraction were prepared as previously published (Lelliott et al., 2005, Medina-Gomez et al., 2005). Real-time PCR was performed using a TaqMan 7900 (Applied Biosystems, http://www.appliedbiosystems.com) according to standard protocols. Primers and TaqMan probes were designed using Primer Express, version 2.0 (Applied Biosystems). Primer and probe sequences are shown in (Suppl. Fig. 2).

### Immunohistochemistry

To characterize the hypothalamic nucleus specific GRP78 expression, we used an immunohistochemical approach with slight modifications as previously described (Camacho, et al., 2007). Antibodies used were primary mouse anti-GRP78 (1:200) and secondary Alexa Fluor 568 donkey anti-mouse IgG (1:1000). Negative controls involved incubating sections with primary or secondary antibodies without the secondary or primary antibody with undetectable signal. Sections were mounted with Vectashield (Vector Laboratories) containing 4′,6-diamidino-2-phenylindole (DAPI) and were analyzed by using an inverted fluoresce microscope (ECLIPSE Ti, Nikon).

### Neuronal culture

Cortical mouse neurons were cultured as described (Papadia, et al., 2005). Cortex was dissected from embryonic E17 pups (1 cortex per culture) in Neurobasal A medium supplemented with B27 (Invitrogen), 1% rat serum, and 1 mm glutamine (Invitrogen) and cultured for 10–14 days as described (Papadia, et al., 2005). ER stress activation was made in trophically deprived medium using thapsigargin (0.5 μM in DMSO), tunicamicyn (4 μM in DMSO) and palmitic acid (40 μM in NaOH 0.1 N) for 1, 3, 6, 12 and 24 h. When required, the role of amino acids on insulin sensitivity was addressed by incubation with (5–500 μM) l-Leucine and 100 nM insulin and/or 50 nM rapamacyn. The effect of mTOR activation on ER stress was evidenced by the stimulation with 0.5 μM thapsigargin and 50 nM rapamacyn. After stimulation, cells were washed with cold phosphate‐buffered saline (PBS) and lysis (150 mM NaCl, 25 mM Tris–HCl pH 7.4, 1% triton, protease inhibitors (04693159001, Roche, UK) and phosphatase inhibitors (04906845001, Roche, UK)/or RLT (RNeasy Mini Kit, Qiagen Ltd, Crawley, UK) buffer were added to perform immunoblot or RT-PCR analysis respectively.

### Flow cytometry analysis of mitochondrial membrane potential

Fresh hypothalamus from WT and PGC-1β KO mice were placed on ice containing PBS and chopped into small pieces, less than 1 mm using blades. Tissue dissociation was performed using serum free Dulbecco's Modified Eagle's Media (DMEM) containing 0.25% trypsin (≥ 6,000 units/mg protein) and incubated at 37 °C for 10 min. After incubation, equal volumes of DMEM containing 10% foetal serum were added to the reaction to disable the enzyme activity. Cell suspension was filtered through a 40 μM cell strainer, followed by 1500 rpm centrifugation for 5 min and the resulting pellet was resuspended in 1 ml of warm PBS. 1 million of hypothalamic cells from WT and PGC-1β mice were used for each assay. To analyze the mitochondrial membrane potential we used 5′,6,6′-tetrachloro-1,1′,3,3′-tetraethyl-benzamidazolocarbocyanin iodide (JC1, M34152 Molecular Probes) following the manufacturer's protocol. This dye can selectively enter the mitochondria, due to its positively charged molecule, where it reversibly aggregates upon membrane polarization causing shifts in emitted light from 530 nm (monomeric form, green fluorescence) to 590 nm (J aggregate form ,red fluorescence) when excited at 488 nm. Briefly, 10 μl of 200 μM JC1 (2 μM final concentration) was added to each sample and incubated at 37 °C, 5% CO_2_ for 15 min. Mitochondrial membrane depolarization was performed by adding 1 μl of 50 mM CCCP (50 μM final concentration) to the cell suspension for 5 min at 37 °C. In these conditions JC1 monomeric aggregate in cytoplasm detected at 530 nm is the predominant form. Mitochondrial membrane depolarization (JC1) was analyzed by flow cytometry on a FACS Calibur and analyzed by Cellquest Pro software (Becton and Dickinson, NJ, United States). The ratio between the fluorescence at 530 nm (monomeric form) to 590 nm (J aggregate form) was calculated as indicative of the mitochondrial membrane depolarization. To adjust the basal values we used hypothalamic neurons from WT mice. In the graph we gave them a value = 1 and we compared the fold of chance in the KO neurons.

### Lipid profiling

Tissue homogenates were prepared and lipids were extracted by the method of Folch et al., 1957 (Folch, et al., 1957), and individual species were measured by ESI-MS/MS using a PE Sciex API 3000 triple quadrupole mass spectrometer. Concentrations are reported in pmol/mg of total cell protein. Relative cholesterol levels are expressed as a ratio of the 20 cholesteryl ester to the 17:0 cholesteryl ester internal standards per mg of total cell protein. For PC and PG species, only the total number of carbons and double bonds of the two fatty acids are reported.

Gangliosides were purified from the aqueous layer using C18 clean up extraction columns. Samples were analyzed on a PE Sciex API 3000 triple quadrupole mass spectrometer with an electrospray source. Ganglioside concentrations were calculated by ratio against a known amount of extracted deuterated ganglioside internal standard. Values are semi-quantitative only. Concentrations are reported in pmol/mg of the total cell protein loaded onto the sucrose gradient for membrane raft isolation. The MRM transition used for this analysis cannot differentiate between C20:1/18:0 and C18:1/20:0. Further fragmentation (i.e. MS3) is proposed in future work and will resolve the species of ganglioside present. However, it has been reported in a number of papers that the most prevalent species in the brain is the C20:1/18:0 species and this is what we have temporarily assigned (Whitfield, et al., 2000), GM1 C18:1/18:0. GM2 and GM3 species have been confirmed by HPLC retention times.

### Statistical analysis

Data analysis was performed using StatView Version 4.5 (Abacus Concepts, Berkley, California, United States). Statistical differences between two groups were evaluated using the unpaired Student's *t* test. Data are presented as mean ± SEM. The significance levels displayed on figures are as follows: * indicates *p* < 0.05.

## Results

### Ablation of PGC-1β leads to brain mitochondrial dysfunction and constitutive activation of mTORC1 pathway

**Fig. 1:**
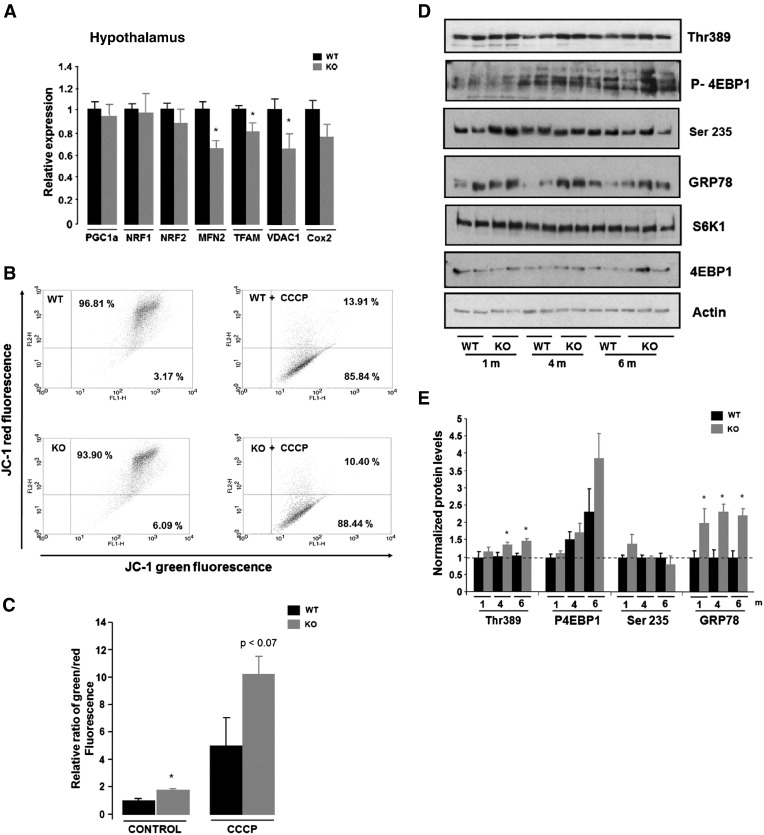
PGC1-β regulates brain mitochondria biogenesis and function. A) Hypothalamic gene expression of mitochondria markers from 8-week-old male WT and PGC1-β KO mice fed chow diet. Individual measurements are standardized using actin, and then the average of the WT group was set to 1. (*n* = 6–8). B–C) Hypothalamic neurons were dissociated as described and basal and stimulated mitochondria activity, using buffer or 1 μl of 50 mM CCCP, respectively, were performed and analyzed by flow cytometry using Alexa Fluor 488 dye and R-phycoerythrin filter. D) Western blot shows the specificity of mTOR core components on Chow diet in WT and PGC1-β KO mice. E) Densitometry analysis of blots for GRP78, p4EBP1, pSer235/236 and Thr389 were corrected against loading control actin, 4EBP1 or S6K1, respectively. Graphs show the normalized results of mean ± SEM for *n* = 6–8 and statistical significance after using unpaired Student's *t* test. *p < 0.05.

Gene expression analyses of hypothalamus from PGC-1β KO compared to WT mice showed a significant decrease in the levels of mitochondrial transcription factor A (TFAM), the voltage dependent anion channel 1 (VDAC1) and mitofusin 2 (MFN2) genes encoding for proteins related to mitochondrial differentiation and mitochondria abundance and mitochondria fusion respectively (Mahad et al., 2009, Scarpulla, 2008) (Fig. 1A). No compensatory upregulation of PGC-1α expression was observed in PGC-1β KO (Fig. 1A). We also observed that hypothalamic neurons from PGC-1β KO mice showed lower membrane potential (lower Δ*Ψ*m) than their WT counterparts and this was evidenced by an increase in the JC1 monomeric form detected at 530 nm, an effect that was consistent in untreated and CCCP treated samples (Figs. 1B and C). Globally, these results suggest that PGC-1β KO neurons may have less mitochondria, less functional mitochondria or a combination of both.

Given that states characterised by impaired mitochondrial function have been shown to inhibit mTOR activation in 3 T3 cell line and also beta cells (Dennis et al., 2001, Desai et al., 2002, Inoki et al., 2003, Kim et al., 2002), we analyzed whether PGC-1β ablation was associated with impaired mTORC1 activation by measuring phosphorylation of its effectors Thr 389 (S6K1), Ser65 (4EBP1) and Ser 235/236 (S6). Unexpectedly, our results showed that under basal conditions, the absence of PGC-1β results in the constitutive activation of mTORC1 pathway in brain, as indicated by the increased phosphorylation of S6K1 (Thr 389) and 4EBP1 (Ser65), but not Ser 235/236 in the hypothalamus of young-adult PGC-1β KO mice (Figs. 1D, E). Of note, this increase in the basal levels of mTORC1 pathway activation correlated with the upregulation of GRP78 protein, a marker of ER stress, in adult mice (Figs. 1D, E). Altogether, these data indicate that ablation of PGC-1β results in a decrease in mitochondrial function, which associates with constitutive mTORC1 and ER stress activation in brain under basal conditions.

### Deletion of PGC-1β leads to inefficient induction of ER stress activation in hypothalamus and cortex in response to High Fat Diet (HFD)

**Fig. 2:**
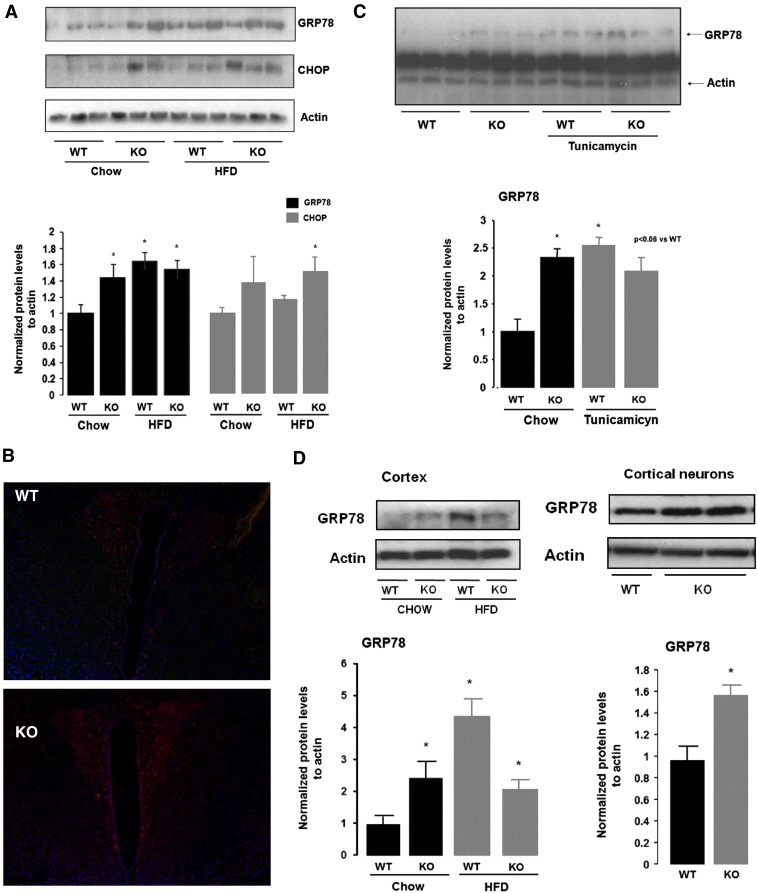
Ablation of PGC1-β results in basal GRP78 expression but inefficient ER stress response on a challenge. A) Western blot shows the specificity of GRP78 and CHOP on Chow and HFD and their densitometry analysis (n = 6 – 8). B) Hypothalamic co-immunostaining for GRP78 (red) and DAPI (blue) in WT and PGC1-β KO mice on Chow diet. C) Western blot shows the specificity of hypothalamic GRP78 and CHOP before and after Tunicamicyn administration (n = 6 – 8). D) Western blot shows the basal increase of GRP78 and inefficient upregulation in the cortex of PGC1-β KO under HFD, and basal GRP78 upregulation in an in vitro system of cortical neurons (n = 6 – 8). Graphs show the normalized results of mean ± SEM and statistical significance after using unpaired Student's t test. *p < 0.05.

Similar to young-adult mice, we observed an increase in the protein levels of GRP78 in older PGC-1β KO mice (8 months old) (Fig. 2A). This result suggests that activation of ER stress is characteristic of ablation of PGC-1β at hypothalamic level. Importantly, immunofluorescent staining and analysis of brains from WT and PGC-1β KO mice indicated that the upregulation of GRP78 in the KO mice is restricted to the paraventricular nucleus (Fig. 2B), a hypothalamic region involved in the coordination of energy homeostasis through its differential sympathetic output to target organs (Pyner, 2009). No changes at gene expression level of GRP78 or additional ER stress markers, such as CHOP, XBPs, and GADD34, were detected between WT and KO when fed chow diet (Suppl. Fig. 3A), suggesting that the basal ER stress activation observed in PGC-1β KO mouse is primary regulated at post-transcriptional level.

**Fig. 3:**
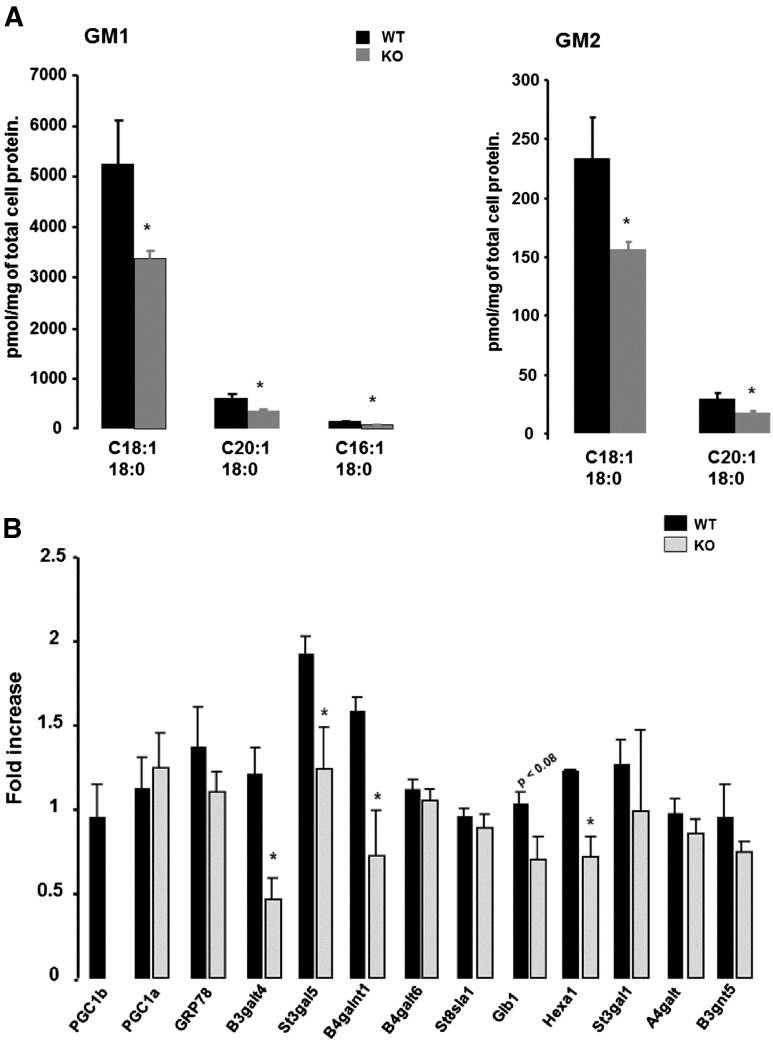
GM1 and GM2 gangliosides levels in the hypothalamus of WT and PG1beta KO mouse. Individual lipid species were measured as described in Materials and methods. Statistical analysis was performed by using a two tail ANOVA following by a post-hoc Bonferroni test. Values are mean 5 standard error of mean. *p < 0.05 versus WT.

It is well established that fatty acid overload induced by mitochondrial dysfunction and/or HFD feeding leads to ER stress in different cell types (Hotamisligil, 2010). Firstly we analyzed if basal activation of ER stress response correlates with hypothalamic lipid accumulation related to mitochondria dysfunction in the PGC-1β KO model. In particular, we explored the role of ceramide species, as it has been demonstrated to induce ER stress (Lang, et al., 2011). Lipid profile of hypothalamic KO mice showed no major changes in ceramides, glucosylceramides, lactosylceramides, phosphoinositides, phosphotidylethanolamines, sphingosine and cholesterol (Suppl. Data), suggesting that ER stress response activation in PGC-1β KO is not related to ceramide accumulation under basal conditions. Of interest, we found a specific reduction in gangliosides species, GM1 (C18:1, 18:0; C20:1, 18:1; C16:1, 18:0) and GM2 (C18:1, 18:0; C20:1, 18:0) (Fig. 3A), which correlates with defects in enzymes regulating this pathway, including B3gal5, B4gal6 and Hexa 1 (Fig. 3B). These results suggest a selective regulation of ganglioside pathway in the brain of PGC-1β KO which may contribute to the ER stress regulation, as we will discuss later.

Additionally, we found that WT fed HFD showed increased hypothalamic GRP78 protein levels in comparison to chow fed WTs, but not in the PGC-1β KO (Fig. 2A). These results suggest that the ablation of PGC-1β prevented further HFD-induced ER stress response. Of note, we also observed an increase in the protein levels of CHOP, a second ER stress and apoptotic cell death marker; in hypothalamus of PGC-1β KO mice fed HFD (Fig. 2A). At first, the latter suggested that this inappropriate ER stress response may be associated with increased susceptibility to neuronal damage and death in PGC-1β KO mice.

The inefficient upregulation of GRP78 in PGC-1β KO mice under ER stress conditions was further confirmed *in vivo* in animals treated intraperitoneally with one dose of tunicamycin, a pharmacological inducer of ER stress for 24 h (Fig. 2C). Specifically, WT but no PGC-1β KO mice treated with tunicamycin showed an increase in hypothalamic GRP78 protein levels as compared to their untreated group (Fig. 2C). We also validated the increase gene expression of GRP78, XBPs, CHOP and GADD34 under this strong pharmacological stimulus in WT hypothalamus but not in PGC-1β KO mice (Suppl. Fig. 3B). Importantly, the expression level of PGC-1α mRNA, that normally compensated the lack of PGC-1β in peripheral organs, was unchanged in the PGC-1β KO brains (data not shown).

The pattern of hypothalamic dysregulation of GRP78 in PGC-1β KO as compared to WT mice, i.e. higher in chow fed mice and unresponsive to HFD stress was also reproducible in the cortex *in vivo* and also in cortical neurons *in vitro*, respectively (Fig. 2D). All together, these results suggest that different regions of the brain are equally sensitive to metabolic disturbance associated to dysfunctional PGC-1β regarding ER stress response.

### Inefficient pharmacologically induced ER stress response in PGC-1β KO mice seems to contribute to apoptotic neuronal death

**Fig. 4:**
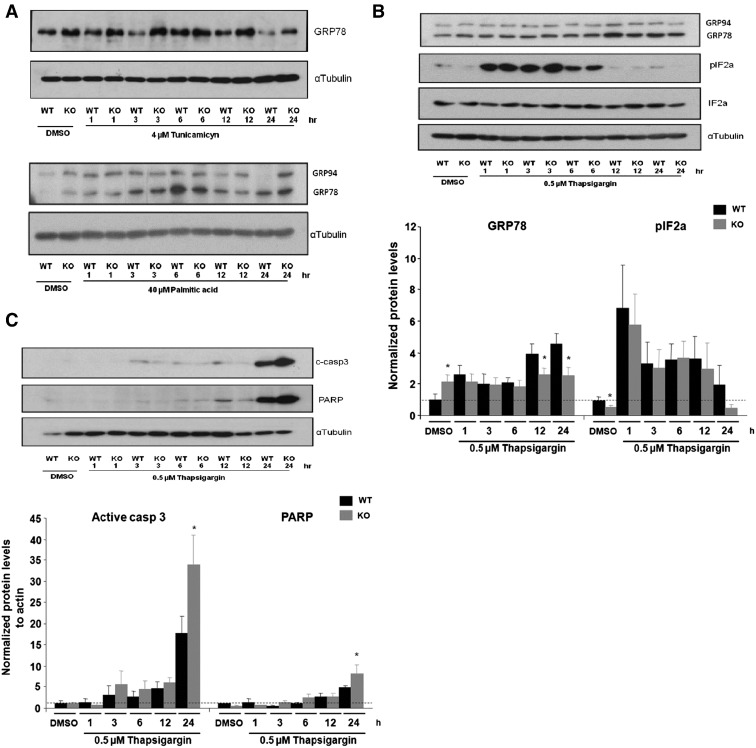
Inefficient ER stress response in PGC1-β KO brain results in neuronal cell death. A) Neuronal culture was stimulated with tunicamycin or palmitic acid as described and protein levels of GRP78 were analyzed by western blot. A representative western is shown. B) Neuronal culture was stimulated with thapsigargin as described and protein levels of GRP78 and pIF2α were analyzed. GRP78 and pIF2a were normalized using total actin and IF2a, respectively. C) Apoptotic markers such as Caspase 3 cleavage and PARP were analyzed after thapsigargin stimulation. Graphs show the normalized results of mean ± SEM for *n* = 5 and statistical significance after using unpaired Student's *t* test. *p < 0.05.

The molecular mechanisms mediating the defective ER stress response in the brains of PGC-1β KO mice under different stress stimulus were further dissected *in vitro*. Mimicking our *in vivo* findings in animals fed a HFD or pharmacologically treated with tunicamycin, *in vitro* treatment of cultured neurons with the ER stress inducers tunicamycin or palmitate caused an increase in GRP78 protein content in wild type at 6 h but not at the same magnitude as PGC-1β KO neurons, when compared with its basal values (Fig. 4A). The antibody can also recognize GRP94 protein, which like GRP78 also has a KDEL sequence (Figs. 4A, B, upper band). Of note, GRP94 protein levels correlate with GRP78 upregulation in our experiments (Figs. 4A, B, upper band), supporting the effect of inefficient ER stress activation in PGC-1β KO neurons. By using a third ER stress inductor, thapsigargin, which inhibits protein glycosylation and promotes protein accumulation in the ER, we also found inefficient GRP78 upregulation in hypothalamic neurons of PGC-1β KO at 12 and 24 h after treatment (Fig. 4B). In terms of gene expression level, no differences in the transcriptional induction of GRP78, CHOP, XBPs and GADD34 were found between WT and PGC-1β KO cultured neurons after our protocol with thapsigargin or tunicamycin treatment (Suppl. Fig. 4). These results reinforce the concept that the inefficient ER response in the PGC-1β KO is primarily coordinated at post transcriptional level.

The discrepancy between the increase in the protein levels and no changes in mRNA level at the basal state in the PGC-1β KO as compared to WT model, could be due to differences in the IF2a phosphorylation, as a candidate for post transcriptional regulation in the basal state between genotypes. Early stages of the ER stress response are characterized by phosphorylation of IF2a resulting in attenuation of protein translation and specific synthesis of GRP78 as a strategy for ER stress relief (Eizirik, et al., 2008). We found that the increase in GRP78 protein levels in the cortical neurons of PGC-1β KO under non‐stimulated basal conditions was associated with decreased IF2a phosphorylation (Fig. 4B). Using the same antibody, we also found increased GRP94 protein levels at the basal state and inefficient upregulation in PGC-1β KO under ER stress challenges, which correlated with GRP78 levels (see Figs. 4A and B, upper band, palmitic and tunicamycin experiment, respectively). Thus, this supported the concept that absence of IF2a phosphorylation increased basal GRP78 levels in PGC-1β KO hypothalamic neurons, an effect that is prevented by IF2a phosphorylation in WT.

Failure to appropriately induce ER stress markers may lead to cell death (Szegezdi, et al., 2006). Hence, we addressed whether the inefficient ER stress response observed in PGC-1β KO neurons increased susceptibility to cellular damage. Stimulation of ER stress response by thapsigargin increased two markers of apoptotic cell death, namely cleaved caspase 3 and its target PARP to a greater extent in PGC-1β KO than in WT neurons (Fig. 4C). Altogether, our data suggest that PGC-1β is necessary for the complete activation of ER stress response and that its inactivation seems to increase the susceptibility to neuronal damage and cell death induced by nutritional and pharmacological ER stressors.

### Constitutive activation of mTORC1 pathway in PGC-1β KO brains determines basal ER stress response

**Fig. 5:**
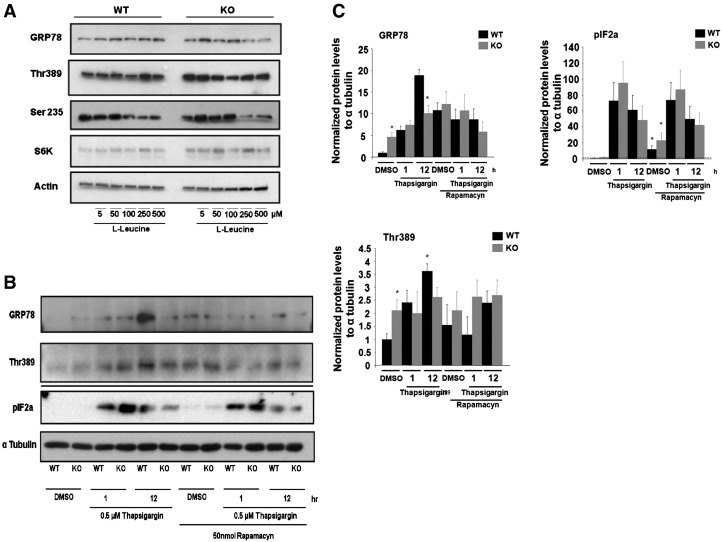
Constitutive mTOR activation in PGC1-β KO model leads to GRP78 expression. A) Neuronal culture was stimulated with l-leucine (5, 50, 100, 250, 500 μM) during 12 h and western blot analysis for GRP78, pThr389 and pSer235/236 was performed. A representative experiment from 3. B, C) Neuronal culture was stimulated with thapsigargin (0.5 μM) or DMSO during 1 or 12 h and protein levels for GRP78, pThr389 and pIF2a was analyzed as described in Fig. 1. For some experiments preincubation with rapamacyn (50 nM) 1 h before and during treatments was performed. Graphs show the normalized results of mean ± SEM for *n* = 3–4 and statistical significance after using unpaired Student's *t* test. *p < 0.05.

It is known that activation of mTOR pathway promotes ER stress (Di Nardo et al., 2009, Inoki et al., 2011, Kato et al., 2012, Ozcan et al., 2008). Thus, we investigated whether the basal ER stress activation observed in PGC-1β KO mice were associated to constitutive activation of the mTOR pathway. Mimicking the *in vivo* studies, our *in vitro* results indicated that PGC-1β KO neurons exhibited activation of mTORC1 in the basal state as evidenced by increased phosphorylation of Thr 389 (S6K1), which correlated with basal upregulation of GRP78 (Fig. 5A).

We next investigated whether nutritional activation of mTOR pathway by branched chain amino acids resulted in differential increase of ER stress markers between genotypes. Thus, *in vitro* activation of mTORC1 with small dose of l-leucine (5, 50 and 100 μM) increased phosphorylation of Thr 389 (S6K1) and Ser 235/236 (S6) and was associated with higher upregulation of GRP78 in PGC-1β KO neurons vs. WT (Fig. 5A). However, at higher concentrations (250 and 500 μM) of branched chain amino acids, the upregulation effects on mTOR and GRP78 caused by l-leucine were lost (Fig. 5A). A priori, these data suggest that ER stress induction in PGC-1β KO is sensitive to the activation by mTOR pathway, recapitulating the constitutive activation of mTOR observed in PGC-1β KO under basal conditions. However, in the context of a strong challenge with branched chain amino acids, PGC-1β KO neurons are not able to further activate the induction of ER stress.

In agreement with our *in vivo* model, cultured neurons *in vitro* from PGC-1β KO showed increased GRP78 protein levels at the basal state and this correlates with Thr 389 phosphorylation (Figs. 5B, C). Activating ER stress response *in vitro* using thapsigargin promoted higher increased Thr 389 (S6K1) phosphorylation and GRP78 upregulation at 12 h in WT and to a lesser extent in PGC-1β KO (Figs. 5B, C). This may suggest that the initial activation of ER stress may also facilitate mTOR activation. Moreover, we also found that during the time course of the ER stress response induced by thapsigargin, PGC-1β KO neurons exhibited lower IF2a phosphorylation than in WT at 12 h (Figs. 5B, C). Finally, we showed that inhibition of mTOR pathway by rapamycin increased basal pIF2a phosphorylation and decreased the upregulation of GRP78 protein levels previously induced by thapsigargin in WT and KO brains at 12 h (Figs. 5B, C). This indicates that mTORC1 pathway may also be activated during ER stress induction in neurons. Together, our results so far indicate that ablation of PGC-1β is associated with constitutive activation of mTORC1 pathway and increased basal GRP78 expression, but that ablation of PGC-1β prevents a further activation of ER stress response under nutritional and pharmacological stimulus.

### HPD feeding promotes mTOR overactivation but inefficient GRP78 expression in brain of PGC-1β KO mice

**Fig. 6:**
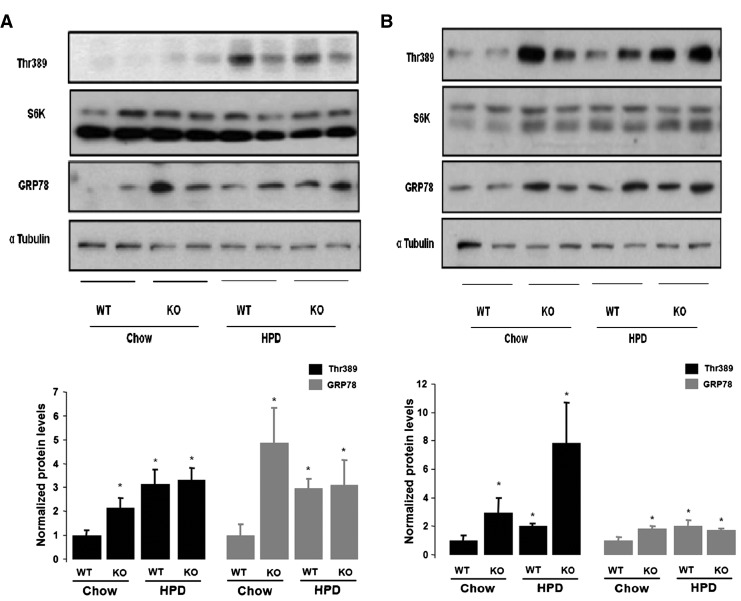
HPD promotes brain mTOR over activation in PGC1-β KO mice. Western blot showing pThr389 and GRP78 expression after HPD feeding in the hypothalamus (A) and cortex (B) of WT and PGC1-β KO mice, respectively. Densitometry analysis is shown below. Graphs show the normalized results of mean ± SEM for *n* = 6–8 and statistical significance after using unpaired Student's *t* test. *p < 0.05.

Next we investigated whether the differences in the activation of mTOR pathway observed between KO and WT *in vitro* upon different doses of l-leucine treatments were recapitulated *in vivo*. We investigated the differential response to hyperproteic and chow diets with respect to activation of mTOR and GRP78 in wild type and PGC-1β KO mice. Our results showed that when fed chow diet, basal Thr 389 (S6K1) phosphorylation and GRP78 protein levels were increased in hypothalamus and cortex of PGC-1β KO mice compared to wild type brains (Figs. 6A, B). The treatment with HPD diet during 14 weeks increased mTORC1 activation, as indicated by increased phosphorylation of Thr 389, similarly in the hypothalamus of both genotypes (Fig. 6A). However, HPD only promoted upregulation of GRP78 in WT but not in PGC-1β KO mice (Fig. 6A). Of note, HPD feeding promoted mTORC1 activation and GRP78 upregulation in the cortex of WT mice and inefficient GRP78 upregulation in PGC-1β KO mice (Fig. 6B). These results suggest that PGC-1β KO is required to mediate the ER stress response induced by nutritional stress via mTOR in the CNS.

## Discussion

Our results support a role of PGC-1β as a coordinator of the expression of mitochondrial function in the brain, in a similar way as it has been reported in other organs such as liver, muscle (Lelliott et al., 2006, Sonoda et al., 2007) and also in neurons *in vitro* (Wareski, et al., 2009). Of note, while mitochondria dysfunction is present in PGC-1β KO neurons under non‐stimulated basal conditions, this is not associated with a wide alteration in lipid species composition including ER stress precursor ceramides. These results suggest that basal ER stress response activation in hypothalamus of PGC-1β KO is unlikely to be related to lipid accumulation. However, we found a decrease in the levels of GM1 and GM2 gangliosides in PGC-1β KO samples which correlates with defective gene expression of enzymes regulating this pathway. Gangliosides have positive effects on ER stress activation by promoting calcium fluxes from ER to mitochondria (Sano et al., 2009, Tessitore et al., 2004). While our results showed gangliosides downregulation, it does not preclude that GM1 and GM2 species decrease might contribute to chances in calcium homeostasis and increase the susceptibility to ER stress activation under chronic nutritional or pharmacological stimulus. In this scenario, it is tentative to propose that defects in gangliosides synthesis may alter the flow of the lipids through the metabolic pathways contributing to disruption in cellular physiology. Supporting our hypothesis, a decrease in phosphatidylethanolamine species promotes disruption in calcium homeostasis resulting in hepatic ER stress and insulin resistance in obesity (Fu, et al., 2011). These results suggest that aberrant lipid metabolism might increase the susceptibility to calcium homeostasis and cellular damage, an open question to be addressed in future research.

Our results also showed that PGC-1β KO neurons seem to be more vulnerable to apoptotic death, as evidenced by the activation of caspase 3 and PARP, a classical apoptotic cascade activated during mitochondrial dysfunction (Szegezdi, et al., 2006) induced by either drugs or nutritional interventions known to activate the ER stress response, such as thapsigargin or HFD, respectively. We have shown that HFD feeding increases brain CHOP protein levels, which is also an ER stress marker, in PGC-1β KO mice when compared to WT, suggesting its contribution to activation of apoptotic cell death under HFD feeding as was reported recently (McNay, et al., 2012), while more experiments have to be done to support this mechanism. Altogether, this indicates that mitochondrial defects in PGC-1β KO neurons appear to increase the susceptibility to neuronal death induced by activation of ER stress response.

The main aim of the present work was to investigate the signalling defects related to the inefficient ER stress activation in PGC-1β KO neurons in response to pharmacological or nutritional challenges. Our data indicate that genetic ablation of PGC-1β is associated with non-stimulated basal activation of mTORC1, as evidenced by S6K1 (Thr389) phosphorylation but not of Ser235/236 of 40S ribosomal protein S6, in adult brains. Ser235/236 phosphorylation might be a downstream target of S6K1 activation (Wullschleger, et al., 2006), but not necessarily activated by this pathway, as was suggested previously (Pende et al., 2004, Ruvinsky et al., 2005). On the other hand, the molecular mechanisms leading to the constitutive activation of mTORC1 pathway in the PGC-1β KO model are unclear. mTORC1 is typically upregulated by four major anabolic regulatory inputs: amino acids, growth factors, ATP/AMP ratio and cytoplasmic calcium (Gulati et al., 2008, Howell and Manning, 2011). Given that glucose and amino acids are metabolized in the mitochondria, it can be hypothesized that basal defects in mitochondria oxidative capacity in the PGC-1β KO model may lead to cytoplasmic nutrient overload and may contribute to the activation of mTORC1, particularly under basal conditions. The potential relevance of this mechanism in the context of obesity is outlined by the fact that plasma amino acid accumulation and mTOR activation has previously been detected in obese humans and *in vivo* animal models of obesity (Felig et al., 1969, Newgard et al., 2009, Tremblay et al., 2007, Wang et al., 2011).

Our study demonstrates that the constitutive mTORC1 activation in the PGC-1β KO mice is associated with increased GRP78 protein levels under basal non‐stimulated conditions. mTOR has been shown to have positive effects on translation promoting the ER stress response (Di Nardo et al., 2009, Inoki et al., 2011, Kato et al., 2012, Ozcan et al., 2008). We speculate that mTOR activation induced by a diet enriched in proteins may exacerbate ER stress in the PGC-1β KO mice. Our *in vitro* studies support this hypothesis by showing that activation of mTOR at low amino acid concentrations (at 5–100 μM l-leucine concentration) in PGC-1β KO neurons lead to increased GRP78 protein levels. However, ablation of PGC-1β compromised the induction of GRP78 in response to higher levels of l-leucine. Similar to the latter response, absence of PGC-1β prevented the expected upregulation of GRP78 in response to chronic increase in plasma amino acid availability, such as what happened with HPD feeding during 14 weeks. While we have not measured the amino acid concentrations in the samples of PGC-1β KO mice after HPD feeding, a recent study using the same dietary conditions showed a threefold increase in plasma l-leucine concentration in mice (Mogami, et al., 2009). Additionally, our data also indicate that *in vitro* pharmacological activation of ER stress by thapsigargin leads to mTOR activation and induction of GRP78 in WT mice, however, it is defective in PGC-1β KO neurons. Altogether our data suggest a failure of mTOR activation by amino acids or ER stress activators to further upregulate GRP78 in the brain of PGC-1β KO mice.

Our present results support the notion that the effect of mTOR inducing a full response of GRP78 under stimulated conditions requires PGC-1β. Together, we could speculate that defects in PGC-1β are associated with basal unstimulated metabolic stress associated with increased mTOR activation which is able to induce a subtle state of ER stress. However, under stimulated stress conditions, the induction of a proper ER stress response requires PGC-1β. The present research also gives us information to suggest that failure to induce an appropriate ER stress response seems to be associated with increased susceptibility to apoptotic neuronal death and further activation of mTOR, probably in an attempt to optimise the ER stress response. In any case, more research is needed to support this hypothesis.

Our *in vivo* and *in vitro* data also provide information that failure to induce GRP78 in the PGC-1β KO mice in response to nutritional or pharmacological challenges may be preferentially coordinated at post-transcriptional level. In support of this we found decreased pIF2a levels in PGC-1β KO brains, both under basal and after stimulation with ER stress inducers. It is known that the PERK/pIF2a pathway prevents ER stress by inhibition of protein translation and by promoting GRP78 synthesis (Eizirik, et al., 2008). The fact that PGC-1β KO brains have activation of mTORC1/S6K1 pathway coupled with decrease of the PERK/pIF2a cascade suggests that the basal increase in GRP78 under non‐stimulated conditions is likely to be related to an increase in the protein(s) translation and its import to the ER facilitated by the activation of both mechanisms. In fact, Kato et al. (2012) demonstrated that mTOR activation promotes ER stress response, a molecular mechanism likely to be involved in our *in vivo* and *in vitro* model to explain the full ER stress response induction under pharmacological or nutritional challenges (using thapsigargin or HPD, respectively) in PGC-1β KO neurons. To our knowledge, this is the first evidence supporting the role of PGC-1β contributing to the ER stress response involving mTORC1 core.

In summary, our data indicate that ablation of PGC-1β causes metabolic stress leading to a mild ER stress response associated to mTOR activation. However, PGC-1β is required for a full appropriate ER stress neuronal response to nutritional and pharmacological stimulus mediated by mTORC1 activation. Absence of PGC-1β seems to increase the susceptibility to neuronal damage mediated by pharmacological stressors.

The following are the supplementary data related to this article.

Suppl. Fig. 1 Antibodies.

Suppl. Fig. 2 Primers.

Suppl. Fig. 3 Ablation of PGC1-β results in inefficient ER stress response on a challenge. A) Real time gene expression of hypothalamic GRP78, XBPs, CHOP and GADD34 in WT vs PGC1-β KO on Chow and HFD (*n* = 6–8). B) Real time gene expression of hypothalamic GRP78, XBPs, CHOP and GADD34 in WT vs PGC1-β KO before and after Tunicamicyn administration in mice. Graphs show the normalized results by actin loading housekeeping gene of mean ± SEM for *n* = 6–8 and statistical significance after using unpaired Student's *t* test *p < 0.05.

Suppl. Fig. 4 Gene expression induction of ER stress response in PGC1-β KO and WT brains. Neuronal culture was stimulated with thapsigargin or tunicamycin during 1, 3, 6, 12 and 24 h and the expression of GRP78, CHOP, GADD34 and XBPs was analyzed by RT-PCR. Graphs show the normalized results by actin loading housekeeping gene of mean ± SEM for *n* = 4 and statistical significance after using unpaired Student's *t* test.

Suppl. Data Lipid species levels in the hypothalamus of WT and PG1beta KO mouse. Individual lipid species were measured as described in Materials and methods. Statistical analysis was performed by using a two tail ANOVA following by a post-hoc Bonferroni test. Values are mean 5 standard error of mean. *p < 0.05 versus WT.
