# Corrigendum to “Ablation of PGC1 beta prevents mTOR dependent endoplasmic reticulum stress response” [Exp. Neurol. 237/2 (2012) 396–406]

**DOI:** 10.1016/j.expneurol.2012.10.003

**Published:** 2013-01

**Authors:** Alberto Camacho, Sergio Rodriguez-Cuenca, Margaret Blount, Xavier Prieur, Nuria Barbarroja, Maria Fuller, Giles E. Hardingham, Antonio Vidal-Puig

**Affiliations:** a University of Cambridge Metabolic Research Laboratories, NIHR Cambridge Biomedical Research Centre Institute of Metabolic Science, Addenbrooke's Hospital, Cambridge, UK; b Hospital Virgen de la Victoria, Málaga, CIBER Fisiopatología de la Obesidad y Nutrición, Instituto de Salud Carlos III, Spain; c University of Edinburgh, Centre for Integrative Physiology, Edinburgh, UK; d Lysosomal Diseases Research Unit, SA Pathology at Women's and Children's Hospital, North Adelaide, SA 5006, Australia

The authors regret that the following Acknowledgment was inadvertently left out of the published article:

PGC1b^−/−^ mice were generated and generously provided by the AstraZeneca Transgenics & Comparative Genomics Department, Mölndal, Sweden.

