# Supplementary material for: Ablation of PGC1 beta prevents mTOR dependent endoplasmic reticulum stress response
Source: Exp Neurol. 2012 Oct;237(2):396–406. doi: 10.1016/j.expneurol.2012.06.031 (PMC3549498; doi:10.1016/j.expneurol.2012.06.031)
Supplement: Suppl. Fig. 1 — Antibodies. [file mmc1.ppt]

## Slide 1
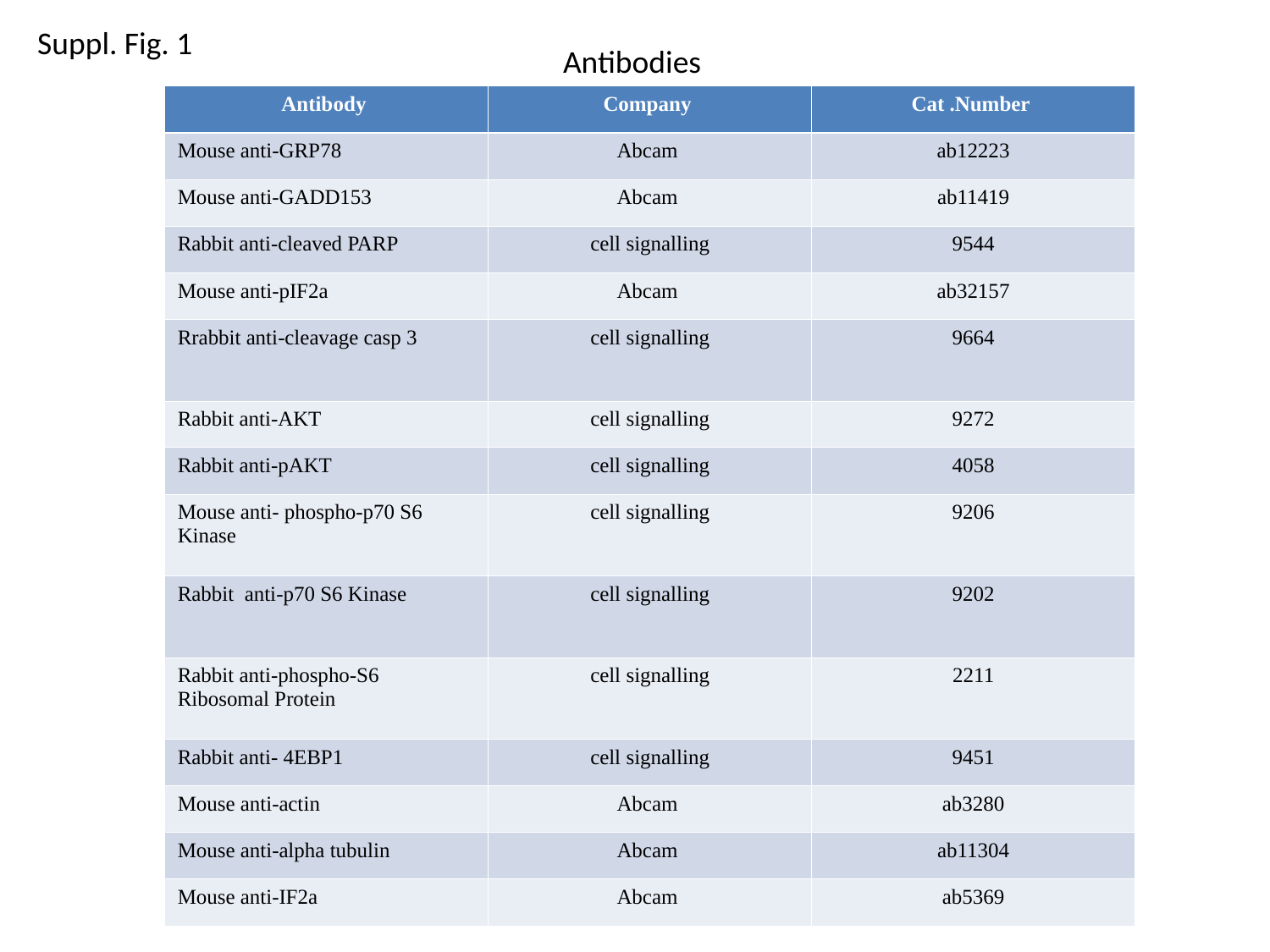

Suppl. Fig. 1
Antibodies
| Antibody | Company | Cat .Number |
| --- | --- | --- |
| Mouse anti-GRP78 | Abcam | ab12223 |
| Mouse anti-GADD153 | Abcam | ab11419 |
| Rabbit anti-cleaved PARP | cell signalling | 9544 |
| Mouse anti-pIF2a | Abcam | ab32157 |
| Rrabbit anti-cleavage casp 3 | cell signalling | 9664 |
| Rabbit anti-AKT | cell signalling | 9272 |
| Rabbit anti-pAKT | cell signalling | 4058 |
| Mouse anti- phospho-p70 S6 Kinase | cell signalling | 9206 |
| Rabbit anti-p70 S6 Kinase | cell signalling | 9202 |
| Rabbit anti-phospho-S6 Ribosomal Protein | cell signalling | 2211 |
| Rabbit anti- 4EBP1 | cell signalling | 9451 |
| Mouse anti-actin | Abcam | ab3280 |
| Mouse anti-alpha tubulin | Abcam | ab11304 |
| Mouse anti-IF2a | Abcam | ab5369 |
