# Supplementary figures and images for: Ablation of PGC1 beta prevents mTOR dependent endoplasmic reticulum stress response
Source: Exp Neurol. 2012 Oct;237(2):396–406. doi: 10.1016/j.expneurol.2012.06.031 (PMC3549498; doi:10.1016/j.expneurol.2012.06.031)

## Slide 1
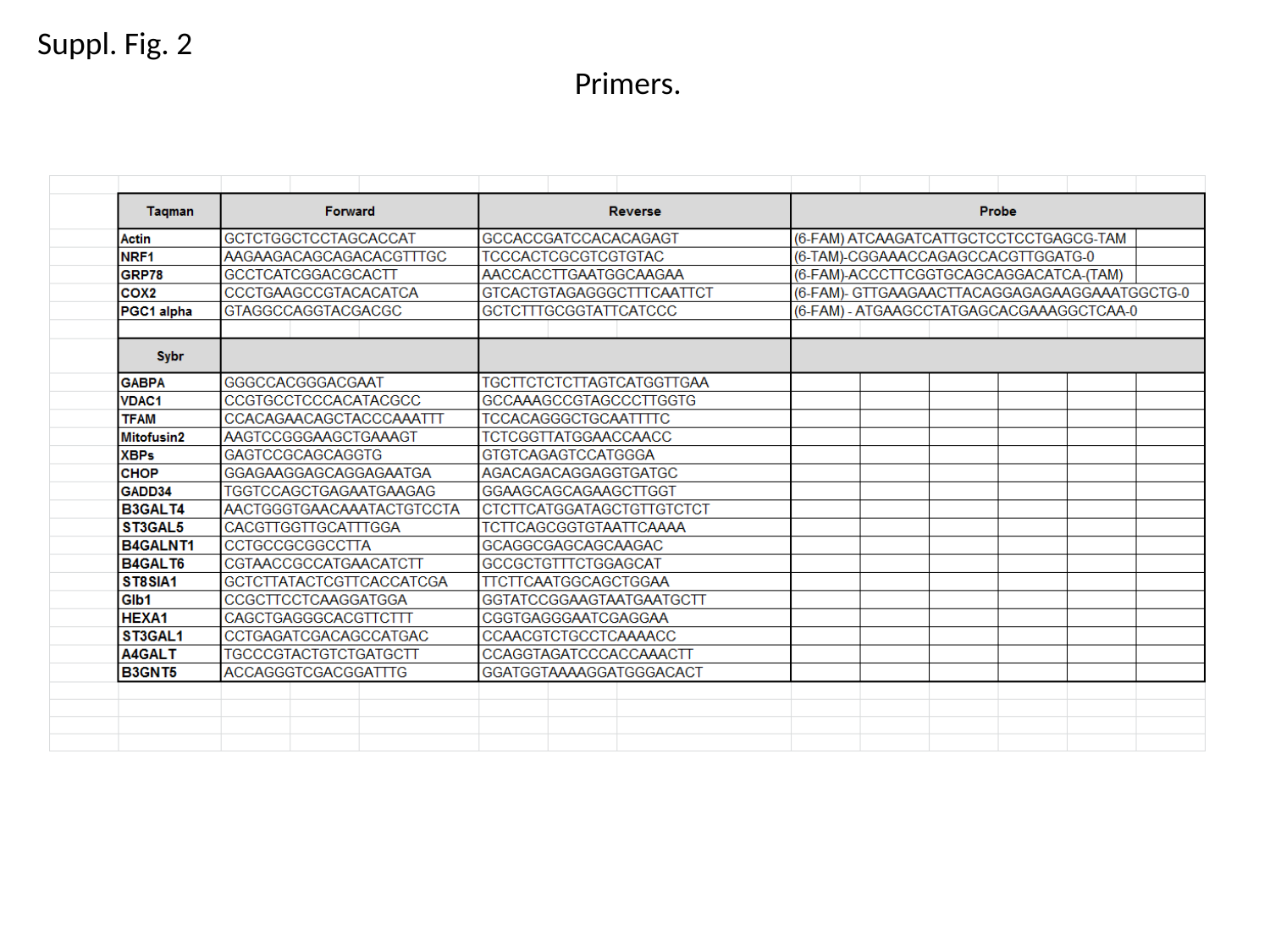

Suppl. Fig. 2
Primers.

Supplement: Suppl. Fig. 2 — Primers. [file mmc2.ppt]

## Slide 1
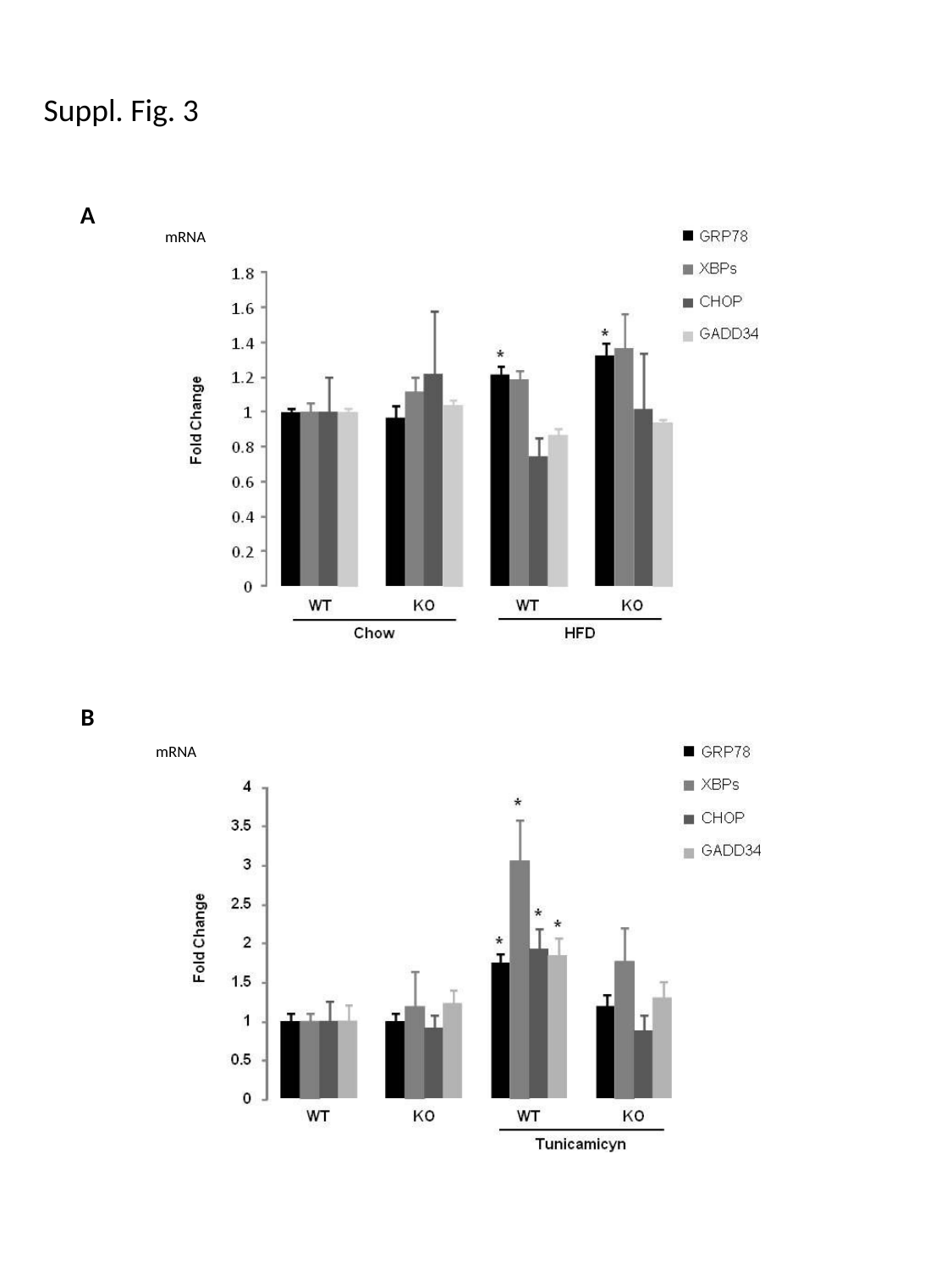

Suppl. Fig. 3
A
mRNA
B
mRNA

## Slide 2
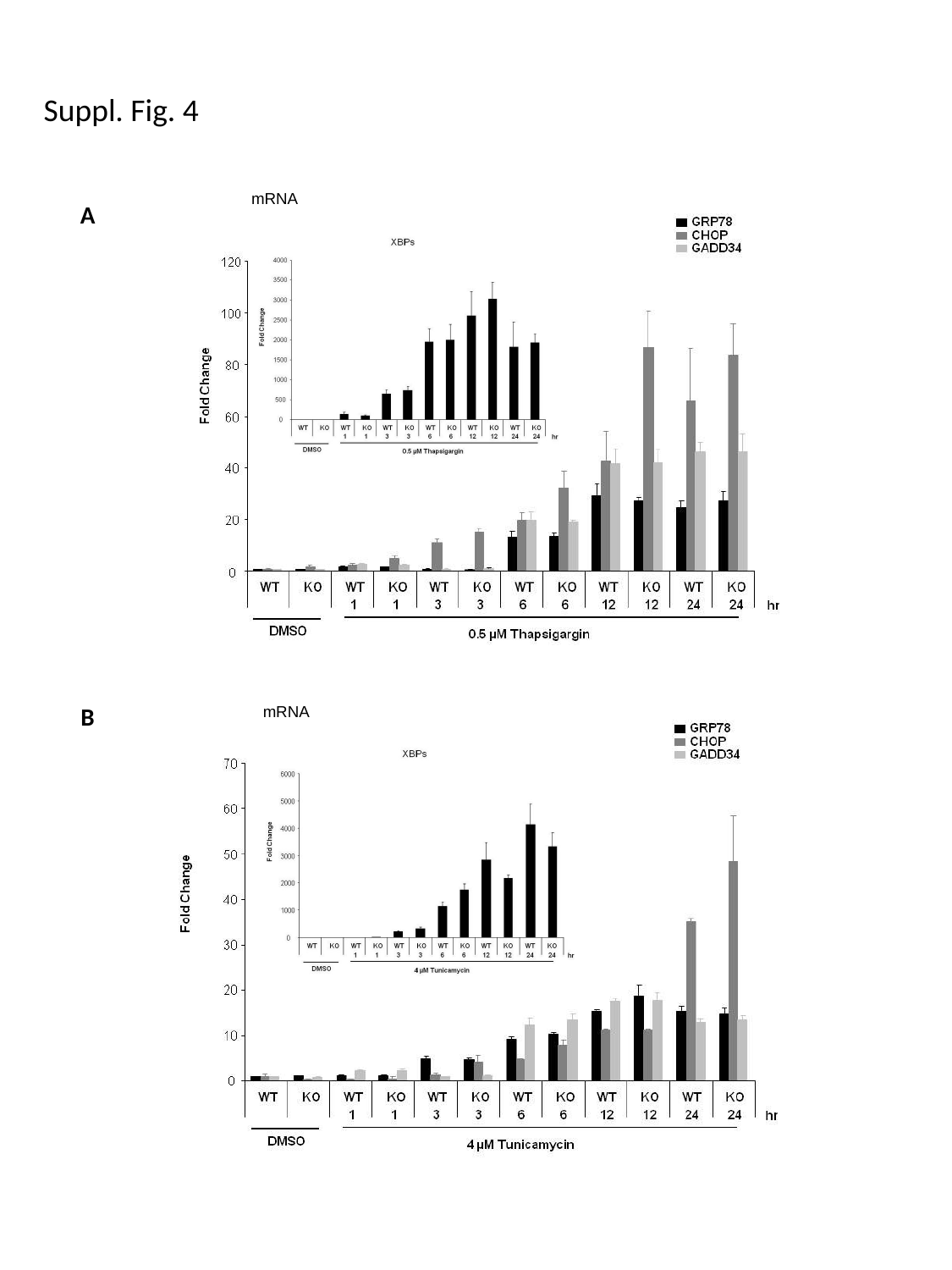

Suppl. Fig. 4
mRNA
A
B
mRNA

Supplement: Suppl. Fig. 3 — Ablation of PGC1-β results in inefficient ER stress response on a challenge. A) Real time gene expression of hypothalamic GRP78, XBPs, CHOP and GADD34 in WT vs PGC1-β KO on Chow and HFD (n = 6–8). B) Real time gene expression of hypothalamic GRP78, XBPs, CHOP and GADD34 in WT vs PGC1-β KO before and after Tunicamicyn administration in mice. Graphs show the normalized results by actin loading housekeeping gene of mean ± SEM for n = 6–8 and statistical significance after using unpaired Student's t test *p < 0.05. [file mmc3.ppt]

Suppl. Fig. 4

A

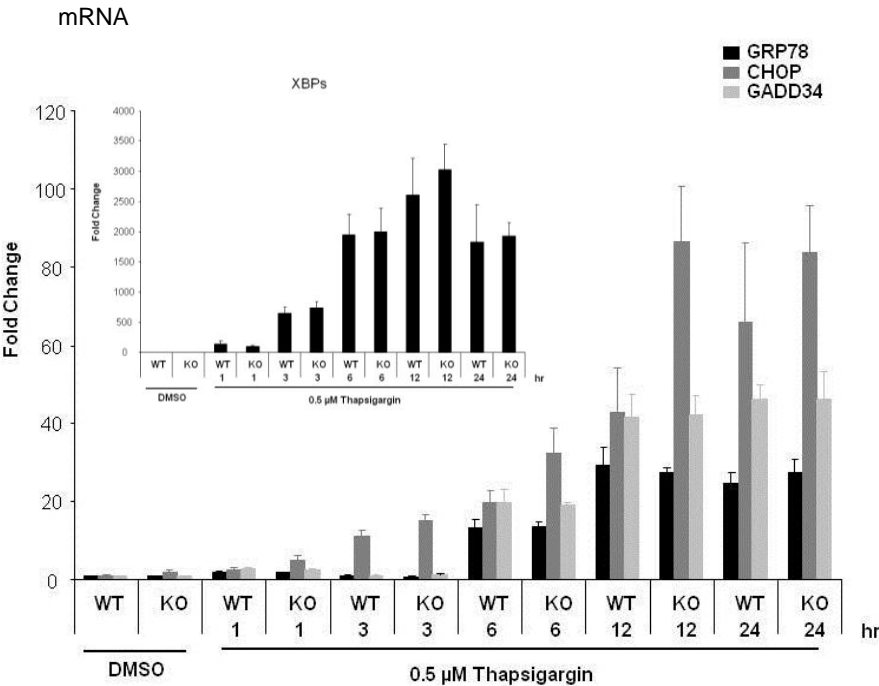

B

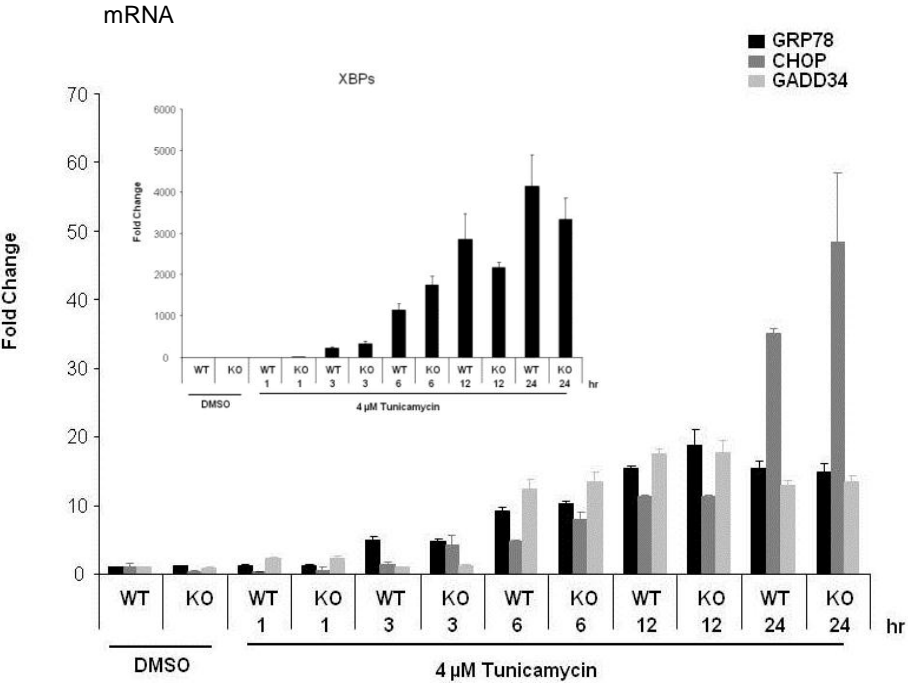

Supplement: Suppl. Fig. 4 — Gene expression induction of ER stress response in PGC1-β KO and WT brains. Neuronal culture was stimulated with thapsigargin or tunicamycin during 1, 3, 6, 12 and 24 h and the expression of GRP78, CHOP, GADD34 and XBPs was analyzed by RT-PCR. Graphs show the normalized results by actin loading housekeeping gene of mean ± SEM for n = 4 and statistical significance after using unpaired Student's t test. [file mmc4.pdf]
